# Alterations in Arginase Activity and Polyamine Metabolism Contribute to the Mode of Action of Canavanine in Tomato Roots

**DOI:** 10.3390/biology15171541

**Published:** 2026-09-04

**Authors:** Pawel Staszek, Katarzyna Ciacka, Agnieszka Gniazdowska

**Affiliations:** Department of Botany and Plant Physiology, Institute of Biology, Warsaw University of Life Sciences, Nowoursynowska 159, 02-776 Warsaw, Poland; katarzyna_ciacka@sggw.edu.pl (K.C.); agnieszka_gniazdowska@sggw.edu.pl (A.G.)

**Keywords:** allelopathy, arginine, non-proteinogenic amino acid, ornithine, polyamine oxidase, putrescine, *Solanum lycopersicum* L.

## Abstract

Canavanine (CAN) is a nonproteinogenic amino acid, synthesised by many legumes (e.g., alfalfa, hairy vetch) and acts as a strong inhibitor of root growth. Thus, CAN may be considered as an allelopathic compound, influencing natural and agroecosystems. CAN is a structural analogue of arginine (Arg), so its toxicity results from its primary mode of action due to interference with Arg-dependent processes, especially incorporation into proteins instead of Arg. The aim of the work was to investigate the impact of CAN (10, 50 µM) treatment (24 or 72 h) on Arg catabolism and content of polyamines (PAs) in the roots of tomato (*Solanum lycopersicum* L.) seedlings. In the roots of plants cultured in the presence of CAN for 72 h, whosegrowth was completely inhibited, arginase activity and transcript levels of *ARG1* and *ARG2* were elevated, while ornithine (the product of Arg catabolism by arginase) content was low. CAN application resulted also in decreased total PAs level and increased relative contribution of spermine in the roots of tomato seedlings. CAN-induced alterations in PAs content in the roots were accompanied by changes in transcript levels of genes related to PAs synthesis (*SPMS*, *SPDS*) and PAs catabolism (*PAO1*).

## Simple Summary

Canavanine is a nonproteinogenic amino acid, a structural analogue of arginine, synthesised by many legumes (e.g., alfalfa, hairy vetch) and acts as a strong inhibitor of root growth. Canavanine is considered as an allelopathic compound, influencing natural and agroecosystems. Its toxicity toward various organisms (animals, microbes, and plants) results from its primary mode of action—incorporation into proteins instead of arginine. Arginine is catabolised by arginase to ornithine, which is the precursor of polyamines. The aim of our work was to investigate impact of the treatment for 24 or 72 h with 10 or 50 µM canavanine on arginine catabolism and content of polyamines in roots of tomato seedlings. In roots of plants cultured in the presence of canavanine for 72 h, growth of which was completely inhibited, arginase activity and transcript levels of genes encoding this enzyme (*ARG1* and *ARG2*) were elevated, while ornithine content was low. Exposure of roots to canavanine resulted in decreased total polyamines (putrescine, spermidine, and spermine) concentration and the increased relative contribution of spermine. Canavanine-induced alterations in polyamines content in the roots were accompanied by changes in transcript levels of genes related to polyamines synthesis (*SPMS*, *SPDS*) and catabolism (*PAO1*).

## 1. Introduction

Canavanine (CAN) is a non-proteinogenic amino acid (NPAA) and a structural analogue of arginine (Arg), produced exclusively by many legumes (Fabaceae), where it serves as a nitrogen storage compound. The richest natural sources of CAN are plants of the genera *Dioclea* and *Wisteria*, whose seeds contain CAN up to 17% of dry weight (DW) [1]. Species of the genus *Canavalia* accumulate lower amounts of CAN, typically 2–8% DW in seeds [2]. However, in *Canavalia* spp. the presence of CAN has also been documented in young vegetative tissues, and the metabolism of this NPAA is the best characterised within the family [3]. In alfalfa (*Medicago sativa* L.) seeds, CAN content ranges from 0.6 to 1.8% DW depending on the cultivar, whereas in commercially available alfalfa sprouts, CAN may reach 2.4% DW [4]. Alfalfa seedlings exude CAN into the surrounding environment, where its concentrations range from 3 to 57 µM depending on the growing medium (aqueous solutions, sand cultures, and filter-paper systems) [5].

Hairy vetch (*Vicia villosa* Roth.) is known for producing strong allelochemical—cyanamide [6,7,8], which is synthesised from CAN [9]. The content of CAN in seeds of hairy vetch reaches approximately 0.8% DW. It was reported that CAN released from hairy vetch protoplasts had a stronger phytotoxic effect than cyanamide [10]. An abundance of CAN in rhizosphere soil was shown when hairy vetch was grown, both under field (8 nmol g^−1^ soil) and greenhouse (23 nmol g^−1^ soil) conditions [11]. In vitro experiments with seeds placed above agar medium, allowing only root contact with the substrate, confirmed that the release of CAN originated from hairy vetch roots rather than from seeds or seed surface residues. Thus, CAN meets the definition of an allelopathic compound, influencing natural and agroecosystems [12].

The primary mechanism of CAN toxicity is its ability to replace or compete with Arg in Arg-dependent metabolic pathways, particularly during translation, resulting in the synthesis of aberrant canavanyl proteins with altered structure and function. This phenomenon has been documented in plants, animals, and microorganisms [13,14]. Accumulation of defective CAN-containing proteins induced proteotoxic stress, disrupted enzymatic activities, and compromised cellular structures, ultimately inhibiting growth and impairing cellular metabolism [3,14].

CAN exhibits strong insecticidal activity, severely impairing growth, development, reproduction, and survival in sensitive insects [14,15,16,17]. CAN also acts as an antimetabolite in cancer cells by interfering with Arg metabolism and protein synthesis and by inducing apoptosis [18,19,20], suggesting that some mechanisms of CAN toxicity may be universal across biological systems.

**Figure 1:**
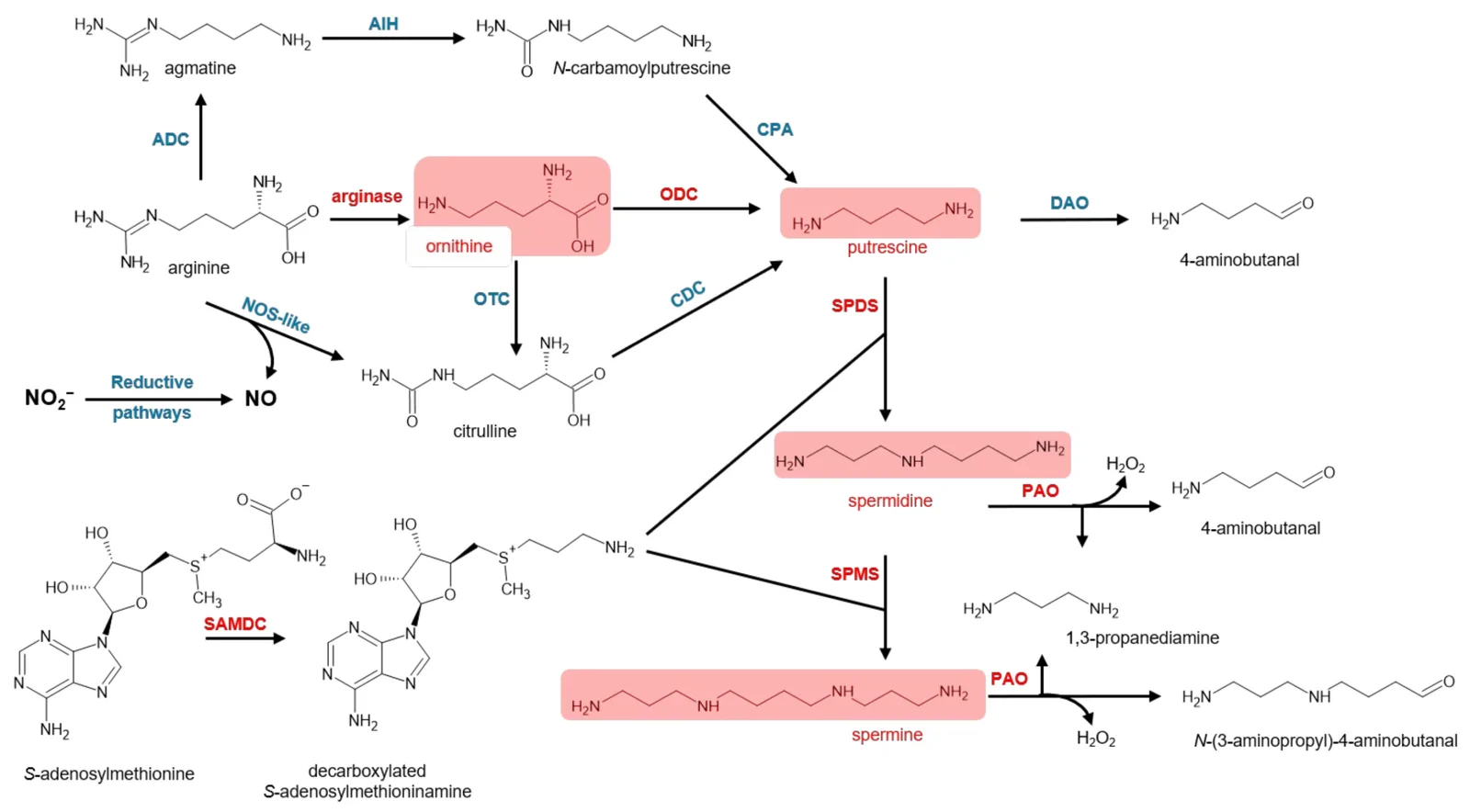
Arginine—polyamines—NO links in plants. Putrescine (Put) is synthesised from arginine (Arg) via agmatine, catalysed by Arg decarboxylase (ADC), and from ornithine (Orn) by Orn decarboxylase (ODC). An additional pathway for Put synthesis depends on citrulline, via the activity of citrulline decarboxylase (CDC). Citrulline originates from Orn by ornithine carbamoyltransferase (OTC) or from Arg as a result of nitric oxide synthase-like (NOS-like) activity. Put is sequentially converted to Spd and Spm through the addition of aminopropyl groups from decarboxylated *S*-adenosylmethionine, formed by *S*-adenosylmethionine decarboxylase (SAMDC). The transfer of the aminopropyl groups is catalysed by Spd and Spm synthases (SPDS/SPMS), respectively. Polyamines catabolism depends on diamine (DAO) and polyamine (PAO) oxidases, which deaminate each PA, producing hydrogen peroxide (H_2_O_2_). Nitric oxide (NO) biosynthesis occurs via reductive pathways using NO_2_^−^. Metabolites and enzymes investigated in this study are marked in red. The structures of the metabolites analysed in the present work are highlighted in pink. Non-investigated enzymes are blue and metabolites are black. ADC, arginine decarboxylase; AIH, agmatine iminohydrolase; CDC, citrulline decarboxylase; CPA, *N*-carbamoyl putrescine amidohydrolase; DAO, diamine oxidases; H_2_O_2_, hydrogen peroxide; NO, nitric oxide (II); NO_2_^−^, nitrite; NOS-like, nitric oxide synthase like enzyme; ODC, ornithine decarboxylase; OTC, ornithine carbamoyltransferase; PAO, polyamine oxidases; SAMDC, *S*-adenosylmethionine decarboxylase; SPDS, spermidine synthase; SPMS, spermine synthase.

Catabolism of Arg depends on the activity of arginase, which is responsible for the hydrolytic decomposition of Arg to ornithine (Orn) and urea [21]. Plant arginase (EC 3.5.3.1) is a manganese-dependent metalloenzyme. Most higher plants typically have one or two arginase isoforms, which have been reported to localise mainly in mitochondria or plastids, and their primary physiological role is linked to nitrogen remobilisation rather than participation in a canonical urea cycle [22]. In CAN-synthesising plants, arginase catalyses the decomposition of CAN to canaline and urea, which is subsequently hydrolysed by urease. In jack bean (*Canavalia ensiformis* (L.) DC), arginase and urease activity in cotyledons and seedlings were coordinated, consistent with a role in nitrogen mobilisation and redistribution [1]. Beyond its primary function in nitrogen remobilisation, plant arginase plays an important regulatory role in stress physiology by modulating Arg flux between competing metabolic pathways that influence reactive oxygen species (ROS) and nitrogen species (RNS) homeostasis (Figure 1) [23]. Arginase catalyses the hydrolysis of Arg, limiting its availability for oxidative production of nitric oxide (NO) and indirectly regulating NO-dependent signalling [24]. Arginase-negative mutants of Arabidopsis (*Arabidopsis thaliana* (L.) Heynh.; *argah1-1* and *argah2-1*) showed altered Arg catabolism, which correlated with NO accumulation (detected using DAF-FM) [24]. Arginase expression was induced by wounding and jasmonate, linking it to biotic stress responses and defence-associated oxidative bursts [25].

In our previous studies using the same experimental model, we demonstrated that in young tomato seedlings, the first visible effect of CAN phytotoxicity was the inhibition of root growth [26]. Root tissues exhibited high sensitivity to this NPAA, with concentrations of 10 and 50 µM resulting in 50% and complete inhibition of root growth, respectively (Figure S1). CAN did not reduce cell viability even under conditions of 100% growth restriction. The negative effects of CAN were mainly noticed in the roots, whereas shoot growth remained unaffected [23,26,27]. We have also shown that CAN may be used as a convenient biochemical tool to modulate NO metabolism in plant cells [27,28]. Although no typical mammalian NOS-like sequence has been identified in plant genomes, Arg-dependent NO production has been confirmed in many plant tissues [29]. It was clearly demonstrated that CAN inhibited Arg-dependent NO production in apple (*Malus domestica* Borkh.) embryos [30] and tomato roots [28], indicating that interference with Arg-dependent NO synthesis represents the primary mode of action of CAN. CAN-induced reduction in NO level was closely associated with the development of secondary oxidative stress in tomato roots [27]. This effect was manifested by increased production of ROS, including superoxide radical (O_2_^•−^) and hydrogen peroxide (H_2_O_2_), as well as by the accumulation of protein carbonyl groups, which are stable markers of oxidative stress. At the same time, no significant oxidative damage to cellular membranes or DNA was observed, suggesting the presence of efficient protective mechanisms limiting the consequences of oxidative stress [23].

CAN, as a structural analogue of Arg, may affect not only NO/RNS production but also the flux of Arg toward Orn and polyamines (PAs) biosynthesis (Figure 1). PAs such as putrescine (Put), spermidine (Spd), and spermine (Spm) belong to plant growth regulators. They are suggested to work as “orthodox-players” modifying growth and development under normal conditions and as “stress-relievers” under biotic and abiotic stresses [31]. Arg is the starting point for PAs synthesis (Figure 1) [32]. It is decarboxylated by Arg decarboxylase (ADC, EC 4.1.1.19) to agmatine, which is a substrate for Put biosynthesis. In some higher plants, including tomato [33], Put is also produced via an alternative pathway involving Orn decarboxylase (ODC, EC 4.1.1.17), which catalyses the decarboxylation of Orn. However, some plant species lack or exhibit reduced ODC activity, so PAs biosynthesis is dependent primarily on the presence of Arg and ADC activity [34]. The existence of an additional pathway of PAs synthesis, demonstrated so far only in sesame (*Sesamum indicum* L.), points to the conversion of Arg to citrulline followed by decarboxylation catalysed by citrulline decarboxylase (CDC), leading to Put generation [35] (Figure 1).

*S*-adenosyl-L-methionine decarboxylase (SAMDC) regulates PAs metabolism and PAs cytosol accumulation (Figure 1). It has already been characterised in different plant species [36], and its activity has been observed in the chloroplast, mitochondria, and cytosol [37]. SAMDC is a rate-limiting, enzyme essential for in Spm and Spd biosynthesis. Its primary function is to catalyse the conversion of *S*-adenosylmethionine (SAM) into decarboxylated SAM (dcSAM), which provides the critical aminopropyl groups required to synthesise Spd and Spm from Put (Figure 1). Deregulation of SAMDC activity severely affected plant development. In potato (*Solanum tuberosum* L.), down-regulation of *SAMDC* resulted in a stunted phenotype with branched stems, short internodes, small leaves, and inhibited root growth [38].

The secondary mode of action of various allelopathic compounds is linked to the modification of phytohormonal balance [39], induction of oxidative stress [40,41] and alteration in the formation of NO and its derivatives [42]. As the most visible reaction of an acceptor plant to allelopathy stress is a restriction in growth and disturbances in development, the role of PAs in plant responses to allelochemicals seems obvious. Despite this, there are very few reports in the literature on the mechanisms of action of allelopathic compounds related to PAs and their metabolism. Indeed, PAs application is described as a treatment used to mitigate the negative effects of allelochemicals or to protect plants against allelopathic stress [43]. Similarly, it is suggested in the improvement of plant tolerance to abiotic stressors [44,45]. However, it remains unclear whether CAN-induced interference with Arg metabolism affects the distribution of Arg between its competing metabolic pathways. In particular, the relationship between arginase activity, Orn formation, and PA metabolism under CAN stress has not been elucidated. Therefore, analysis of these interconnected pathways may provide new insight into the mechanisms underlying CAN-induced inhibition of plant growth.

The aim of our study was to investigate whether CAN, the structural analogue of Arg, affects Arg catabolism (defined as the primary mode of action) and the metabolic pathways of Arg related to the biosynthesis of PAs (understood as the secondary mode of action). In this work, we have focused on the activity of arginase and transcript levels of genes (*ARG1* and *ARG2*) encoding this enzyme in roots of tomato seedlings exposed for 24 or 72 h to (10 or 50 µM) CAN. To associate the CAN mode of action with PAs, the content of Orn, Put, Spd, and Spm was analysed and compared with transcript levels of genes (*ODC*, *SAMDC*, *SPDS*, and *SPMS*) encoding the main enzymes of PAs biosynthetic pathways. Based on our previous data related to the activity of polyamine oxidase (PAO) (responsible for PAs catabolism, acting as an additional source of ROS) [27], and alterations in *PAO1* transcript level and PAs content, we suppose that the CAN-induced inhibition of tomato root growth may be due to this NPAA influence on PAs metabolism, disrupting mechanisms of the plant’s resistance to stresses. Moreover, we have demonstrated that complete restriction in root growth by CAN at a higher concentration (50 µM) after prolonged exposure is associated with severe changes in arginase activity and *ARG1*, *ARG2* transcript levels. We also suggest that although CAN is actively metabolised by arginase in tomato roots, the extent of CAN degradation is insufficient to avoid its toxicity.

## 2. Materials and Methods

### 2.1. Plant Material

Tomato seeds (*Solanum lycopersicum* L. cv. Malinowy Ożarowski, obtained from PNOS Company, Ożarów Mazowiecki, Poland) were germinated in water for 3–4 days at 20 °C in darkness. Seedlings with primary roots approximately 5 mm were selected and transferred to Petri dishes (⌀ 9 cm) lined with filter paper and moistened with 3.5 mL of water (control) or aqueous solutions of CAN (L-enantiomer, C1625, Merck, Darmstadt, Germany). Each Petri dish contained 20 seedlings. Seedlings were treated with CAN at a low dose (10 μM), which inhibited root growth by 50%, and at a high dose (50 μM), which led to complete inhibition of root growth without a lethal effect [27]. Representative images illustrating the effect of CAN on seedling growth are shown in Figure S1. The treatment solutions were replaced after 48 h of the experiment. The pH of water and the CAN solutions was 7.5. The osmotic potential of the tested solutions was for water: −0.07 MPa, 10 μM CAN: −0.11 MPa, 50 μM: −0.13 MPa. The seedlings were cultured in a growth chamber under a 12/12 h day/night regime, with a temperature of 23 °C (day)/20 °C (night) and a light intensity of 100 µmol PAR m^−2^ s^−1^ for 24 and 72 h [28]. The relative humidity was maintained at 70%. Petri dishes were randomly distributed within the growth chamber. Roots were collected in the morning (the beginning of the light period). Roots of the seedlings were pooled from two Petri dishes (approximately 40 randomly selected seedlings) to constitute one biological replicate then frozen in liquid nitrogen and stored at −80 °C for further use.

### 2.2. Arginase Activity Measurement

Frozen roots of tomato seedlings were homogenised in a chilled mortar with extraction buffer (50 mM MOPS (pH 7.2), 2 mM β-mercaptoethanol, 1 mM PMSF, and 0.1% (*v*/*v*) protease inhibitor cocktail (P9599, Merck, Darmstadt, Germany), 2% (*w*/*v*) PVPP), at a ratio of 1 mL of buffer per approximately 100 mg of roots.

The homogenates were transferred to Eppendorf tubes, thoroughly mixed, and centrifuged for 4 min at 1800× *g* at 4 °C. The supernatants were collected, and Triton X-100 was added to a final concentration of 0.5% (*v*/*v*). Samples were gently shaken for 5 min at room temperature (RT) and subsequently centrifuged for 10 min at 20,000× *g* at 4 °C.

The 0.5 mL of supernatants were transferred to concentrators (Pierce Protein Concentrators, 3 kDa MWCO, Thermo Scientific, Waltham, MA, USA) and centrifuged for 30 min at 20,000× *g* at 4 °C to remove low-molecular-weight compounds from the extracts. To 50 µL of the extract (retentate form concentrators), MnCl_2_ was added to obtain a final concentration of 5 mM. Samples were activated by incubation at 56 °C for 10 min.

Arginase activity was determined using a modified Schimke method [46]. The enzymatic reaction mixture consisted of 50 µL of activated extract and 350 µL of CHES buffer (pH 9.6) supplemented with Arg (A5006, Merck, Darmstadt, Germany) to a final concentration of 250 mM. Incubation was carried out for 60 min at 37 °C with gentle shaking. The reaction was terminated by the addition of 400 µL of an acid mixture (H_2_SO_4_:H_3_PO_4_:H_2_O (1:3:7, *v*/*v*/*v*)). To develop the colour product, 25 µL of α-isonitrosopropiophenone (3% *w*/*v* in 99% ethanol) was added. The reaction mixtures were incubated at 96 °C for 45 min with continuous shaking. Then, samples were cooled for 10 min at RT in the dark, and the absorbance was measured at 550 nm. Urea concentration was determined from the standard curve, and arginase activity was expressed as µg urea mg^−1^ protein h^−1^.

Protein concentration was determined using the bicinchoninic acid (BCA, GK3616 Glentham Life Sciences, Corsham, UK) method [47]. The BCA working reagent was prepared by mixing 8% *w*/*v* sodium hydroxide, 1.6% *w*/*v* sodium potassium tartrate, 4% *w*/*v* BCA solution and 4% *w*/*v* copper sulphate solution in a ratio of 25:25:1 (*v*/*v*/*v*). Absorbance was measured at 562 nm. A standard curve was prepared using bovine serum albumin (126575, Merck, Darmstadt, Germany) in the same extraction background.

### 2.3. PAs and Orn Levels Determination

The concentration of PAs and Orn was determined as described by Krasuska et al. [48]. Tomato roots (100 mg) were homogenised in 1 mL of 80% (*v*/*v*) methanol with an internal standard (10 µL of 1 mM α-methyl-L-phenylalanine (286656, Sigma-Aldrich, Darmstadt, Germany)). Homogenates were mixed for 1 min and then centrifuged at 12,000× *g* for 15 min. The supernatants were collected and filtered through KTL syringe filters (nylon, 0.22 µm pore size). After evaporation of methanol, the residues were re-dissolved in 180 µL of 0.2 M borate buffer (pH 8.8) for derivatisation.

For fluorescence derivatisation, 6-aminoquinolyl-*N*-hydroxysuccinimidyl carbamate (AQC, Synchem UG & Co. KG, Felsberg/Altenburg, Germany) was dissolved in acetonitrile to obtain 2 mM solution. Subsequently, 20 µL of the AQC solution was added to each sample, thoroughly mixed, and incubated at RT for 10 min. Afterwards, samples were incubated at 55 °C for an additional 10 min prior to chromatographic analysis.

After cooling, derivatised samples (20 µL) were injected onto a Bionacom Velocity LPH C18 column (4.6 × 150 mm, 3 µm). PAs were detected using a FP-2020/2025 Intelligent Fluorescence Detector (JASCO Corporation, Hachioji, Tokyo, Japan) with excitation at 250 nm and emission at 395 nm. The column temperature was 35 °C, and the flow rate was set to 1 mL min^−1^.

The mobile phase consisted of solvent A (0.15 M acetate buffer, pH 6.8, containing 0.033% (*w*/*v*) triethylamine) and solvent B (acetonitrile). The gradient programme was as follows: 0–7 min, 95–85% A; 7–16 min, 85–75% A; 16–21 min, 75–65% A; 21–26 min, 65–40% A; 26–30 min, 40–10% A; 30–32 min, 10–5% A; 32–60 min, 5–95% A.

Standard curves for Put, Spd, Spm, and Orn were prepared in 0.2 M borate buffer (pH 8.8), derivatised with AQC, and analysed under the same conditions. PAs concentrations were expressed as nmol g^−1^ fresh weight (FW), Orn content as µmol g^−1^ FW. The total PAs content was calculated as the sum of Put, Spd, and Spm. In addition, the relative proportion of each PAs to the total PA pool was calculated.

### 2.4. Gene Expression Analysis

Gene expression analysis was performed in root tissues using quantitative real-time polymerase chain reaction (qRT-PCR). Total RNA was extracted and purified with RNAzol RT (R4533, Sigma-Aldrich, Darmstadt, Germany) according to the manufacturer’s instructions. Approximately 100 mg of frozen root tissue was used for each RNA extraction. Residual genomic DNA was removed by treatment with DNase I (EN0521, Thermo Scientific™, Waltham, MA, USA). RNA concentration and purity were determined using spectrophotometer (NanoDrop^TM^ 2000, Thermo Scientific™, Waltham, MA, USA). First-strand complementary DNA (cDNA) was synthesised from 200 ng of total RNA using the RevertAid First Strand cDNA Synthesis Kit (K1622, Thermo Scientific™, Waltham, MA, USA) with oligo(dT) primers, in a final volume of 35 µL.

qRT-PCR was carried out using a CFX Connect™ Real-Time PCR Detection System (Bio-Rad, Hercules, CA, USA). Each reaction was performed in a total volume of 12 µL containing 6 µL of iTaq™ Universal SYBR^®^ Green Supermix (Bio-Rad, Hercules, CA, USA), 0.5 µL of 10 µM forward primer, 0.5 µL of 10 µM reverse primer, 4 µL of nuclease-free water, and 1 µL of cDNA template. Melting-curve analysis was performed after amplification, and the presence of a single melting peak confirmed amplification of a single product. No-template controls were included and showed no amplification. The thermal cycling programme consisted of an initial denaturation at 95 °C for 1 min, followed by 40 cycles of denaturation at 95 °C for 15 s, annealing at 60 °C for 30 s, and extension at 72 °C for 30 s. After amplification, a final extension at 72 °C for 1 min was performed, followed by melting curve analysis from 65 °C to 95 °C with 0.5 °C increments. A single melting peak was observed for each primer pair, confirming the amplification of a single specific product.

Gene-specific primers were designed using Primer3Plus software (web interface, accessed in 2023). Primer sequences, NCBI accession numbers, amplicon sizes, and PCR amplification efficiencies are provided in Table S1. PCR amplification efficiencies were determined using LinRegPCR software (v. 2021.2) by linear regression analysis of the log-linear phase of individual amplification curves, as described by Ruijter et al. [49].

The stability of five candidate reference genes was evaluated using NormFinder (v. 0.953) across all experimental conditions. EF1α and PP2A were identified as the most stable combination of reference genes (stability value = 0.007) and were therefore selected for normalisation. Normalised relative quantities were calculated using an efficiency-corrected relative quantification approach with normalisation based on the geometric mean of the two reference genes [50]. Untreated samples at the corresponding time point were used as calibrators [28,50].

### 2.5. Statistical Analysis

The exact number of biological replicates (*n*) is indicated in the corresponding figure description and table title. Data were analysed using Statistica software (StatSoft Inc., Tulsa, OK, USA). Mean values ± SD were calculated for all measured parameters. Statistical significance was assessed by two-way analysis of variance (ANOVA), with CAN treatment and treatment duration as factors, followed by Tukey’s HSD post hoc test at *p* ≤ 0.05. For gene expression analysis, statistical tests were performed on log_2_-transformed normalised relative quantities using two-way ANOVA followed by Tukey’s HSD post hoc test at *p* ≤ 0.05. The assumptions of normality and homogeneity of variances were assessed using the Shapiro–Wilk and Levene’s tests, respectively. The corresponding *p*-values are provided in Table S2.

## 3. Results

### 3.1. CAN Increases Arginase Activity in Roots After 72 h of Tomato Plants Treatment and Leads to Elevated Transcript Level of ARG2

**Figure 2:**
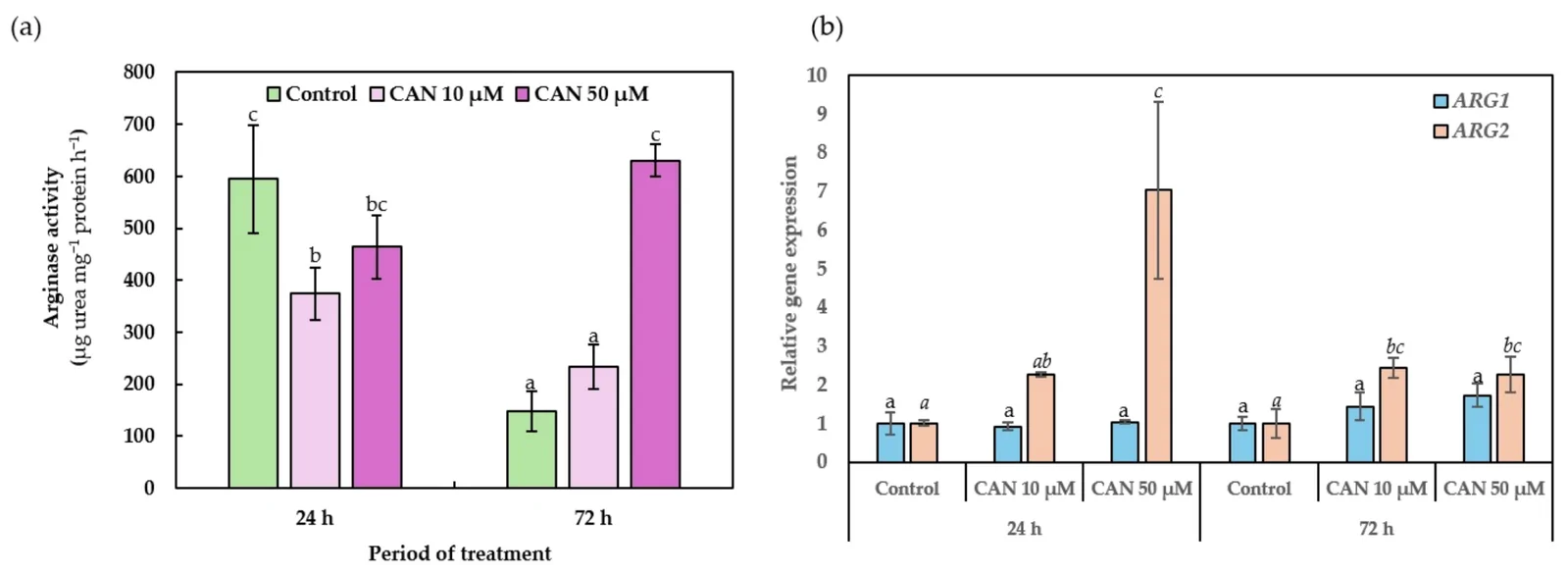
Arginase activity (**a**) and transcript levels of genes encoding arginase (*ARG1* and *ARG2*) (**b**) in roots of tomato seedlings grown in water (control) or treated with 10 or 50 µM CAN after 24 and 72 h of the culture. Values referring to enzymatic activity are means ± SD, n = 5. For *ARG1* and *ARG2*, relative gene expression is presented as means ± SD, n = 4. Data were analysed by two-way ANOVA followed by Tukey’s HSD test. Letters indicate homogeneous groups determined separately for arginase activity and for each gene at *p* ≤ 0.05.

Arginase activity in extracts from the roots of control tomato seedlings after 24 h of cultivation was approximately 600 µg urea mg^−1^ protein h^−1^ (Figure 2a). The activity of this enzyme in tomato roots was significantly influenced by CAN, depending on both the concentration of this NPAA and the duration of the treatment. Supplementing plants with CAN for 24 h led to a significant decrease in arginase activity. The inhibition was more pronounced in extracts from the roots of seedlings treated with 10 µM CAN. Extracts from seedlings exposed to a higher CAN dose showed a decrease of only about 20–25%.

After 72 h of culture, arginase activity in extracts from roots of untreated control seedlings reached about 150 µg urea mg^−1^ protein h^−1^. Arginase activity in roots of seedlings treated with 10 µM CAN was slightly higher than in the control, but this difference was not statistically significant. The strongest stimulation of enzyme activity was observed in the extracts from roots of seedlings treated with CAN at the higher concentration for 72 h, in which arginase activity was more than four-fold higher than in extracts from roots of control plants (Figure 2a).

Transcript levels of the two tomato arginase genes, *ARG1* and *ARG2*, were analysed in the roots of tomato seedlings after 24 and 72 h treatment with CAN (Figure 2b). After 24 h, *ARG1* transcript levels remained close to the control in the roots of seedlings treated with CAN, regardless of the CAN concentration. In contrast, *ARG2* transcript level was strongly induced already after short-term exposure to CAN. In the roots of plants supplemented with 10 µM CAN, *ARG2* transcript levels were more than one relative unit higher, whereas treatment with 50 µM CAN resulted in a six-fold increase in *ARG2* transcript level compared with the control (Figure 2b).

After 72 h, transcript levels of *ARG2* remained elevated irrespective of CAN concentration (Figure 2b).

### 3.2. CAN Increases Orn Content in Tomato Roots

**Figure 3:**
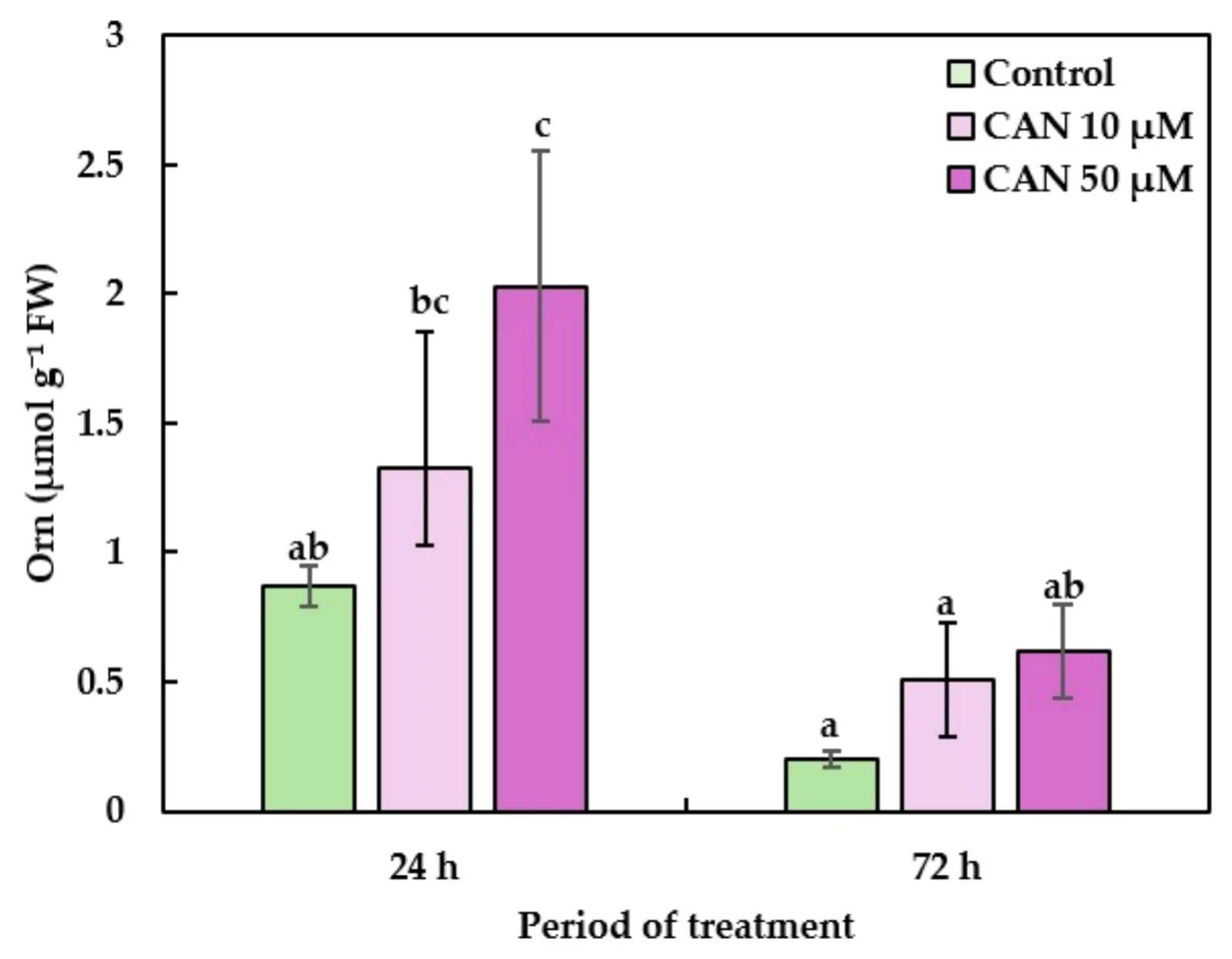
Concentration of Orn in roots of tomato plants grown in water (control) or treated with 10 or 50 µM CAN after 24 and 72 h of the culture. Values are means ± SD, n = 4. Letters indicate homogenous groups determined by two-way ANOVA and Tukey’s HSD test at *p* ≤ 0.05.

In control seedlings, Orn concentration after 24 h of the culture reached nearly 1 µmol g^−1^ FW. After 72 h of the culture, Orn was only approximately 0.25 µmol g^−1^ FW (Figure 3). Short-term exposure to CAN promoted Orn accumulation in tomato roots. CAN (10 µM) slightly increased the Orn level compared with the control; an even stronger effect was observed at 50 µM CAN. After 72 h of CAN supplementation, the overall decrease in Orn content was observed. The concentration of Orn in roots of plants exposed to CAN was around 0.5 µmol g^−1^ FW (Figure 3).

### 3.3. Short-Term (24 h) Treatment of Tomato Plants with 50 µM CAN Up-Regulates Expression of Genes Related to PAs Biosynthesis (SAMDC, SPDS, SPMS)

**Figure 4:**
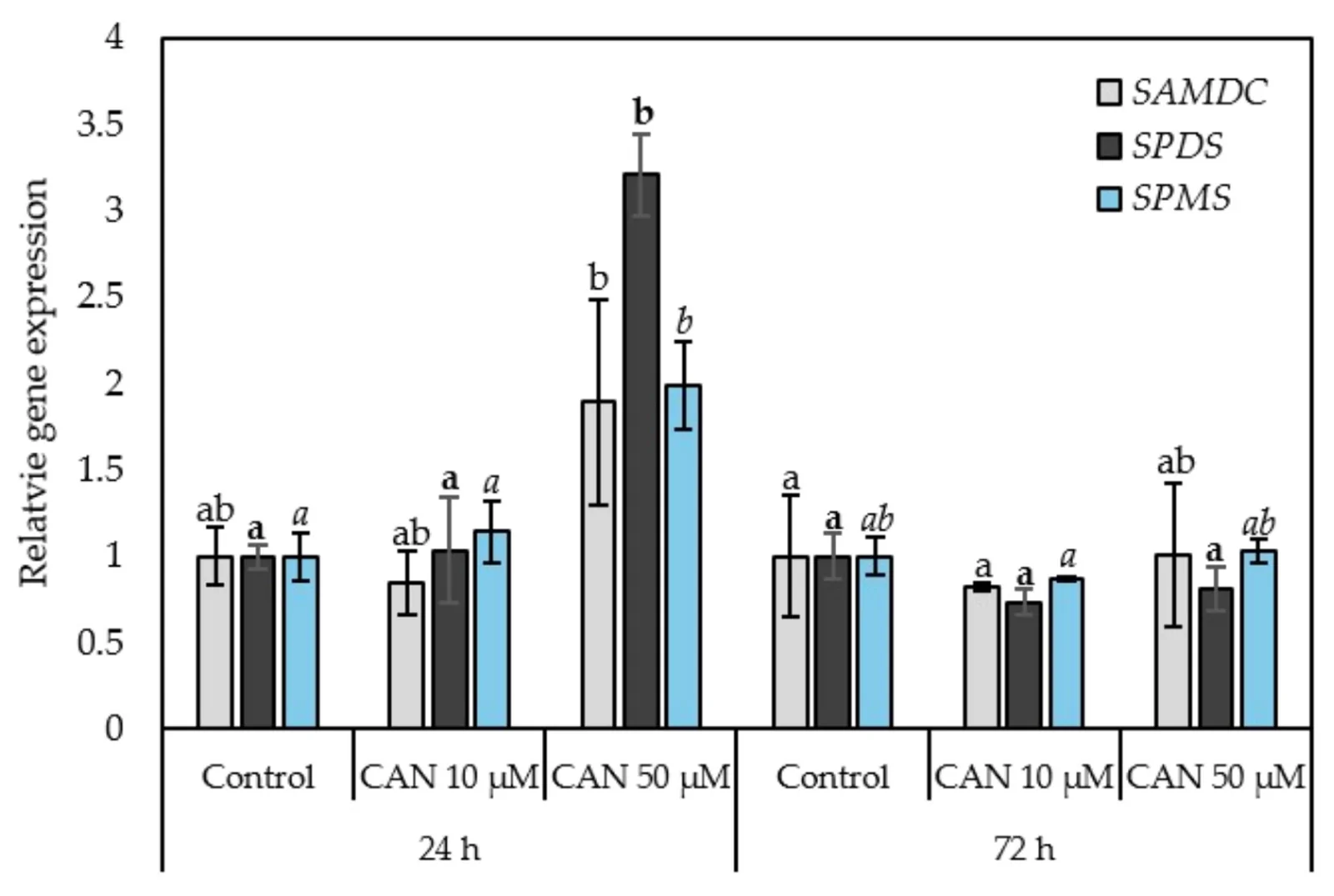
Expression levels of genes encoding enzymes related to PAs biosynthesis (*SAMDC*, *SPDS*, *SPMS*) in the roots of tomato plants grown in water (control) or treated with 10 or 50 µM CAN after 24 and 72 h of the culture. Relative gene expression is presented as means ± SD, n = 4. Data were analysed by two-way ANOVA followed by Tukey’s HSD test. Letters indicate homogeneous groups determined separately for each gene at *p* ≤ 0.05.

The transcript level of *SAMDC* did not differ significantly from the control, regardless of CAN concentration and the duration of NPAA stress (Figure 4).

The transcript level of *SPDS* increased significantly in tomato roots exposed to 50 µM CAN for 24 h; reaching the highest relative transcript level among the analysed genes (Figure 4). No statistically significant differences were detected in the transcript levels of *SPDS* after 72 h of plants culture with CAN.

Transcript levels of *SPMS* were also significantly higher after 24 h of the treatment with 50 µM CAN, while remaining unchanged after exposure to 10 µM CAN. The expression of *SPMS* did not differ significantly from the control after 72 h of the treatment, irrespective of the CAN concentration applied (Figure 4).

### 3.4. Prolonged Treatment with CAN Lowers Total PAs Content in Roots of Tomato Seedlings and Increases the Contribution of Spm in PAs Pool

During the culture period, the total PAs pool in the roots of control plants increased from about 300 after 24 h to 400 nmol g^−1^ FW after 72 h (Table 1).

Short-term (24 h) application of 10 µM CAN resulted in transient accumulation of total PAs in roots, while at the same time 50 µM CAN induced a slight decline in total PAs concentration to about 230 nmol g^−1^ FW as compared to the control (Table 1).

After 72 h, CAN treatment resulted in a marked decrease in total PAs content in roots (Table 1).

Analysis of the relative composition of the PAs pool revealed that Put and Spd were predominant in tomato roots (Table 1). During the whole experimental period in control seedlings, Put reached about 60% and Spd 37% of the total PA, whereas Spm was less than 2% of the total PAs pool. After 24 h of CAN exposure, the relative contribution of Spm was nearly 6% at both CAN concentrations. Later, contribution of Spm in CAN-stressed roots decreased slightly but was two-fold higher than in the control seedlings (Table 1). The Put/Spd ratio increased slightly under CAN supplementation.

**Figure 5:**
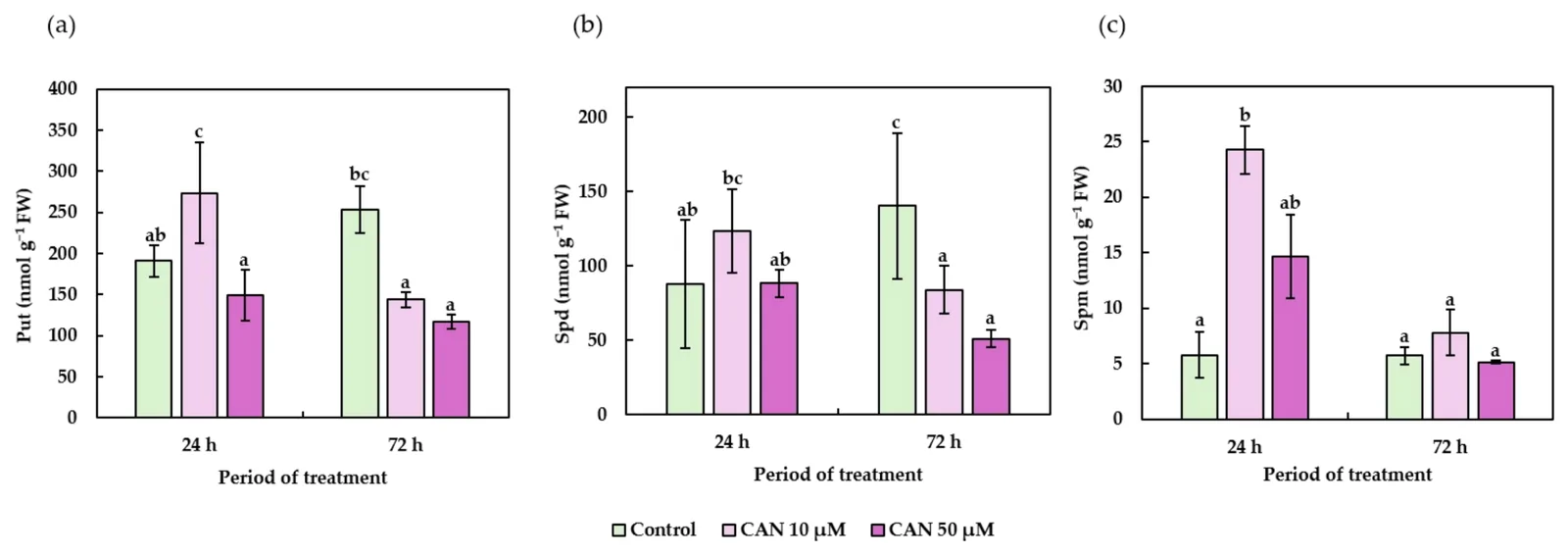
PAs (Put, Spd, Spm) concentration in roots extracts of tomato seedlings grown in water (control) or treated with 10 or 50 µM CAN after 24 and 72 h of the culture. Values are average ± SD, n = 4. Letters (**a**–**c**) indicate homogenous groups determined by two-way ANOVA followed by Tukey’s HSD test at *p* ≤ 0.05.

The concentration of individual PAs: Put, Spd, and Spm in roots of tomato plants grown in 10 or 50 µM CAN is shown at Figure 5.

Put content was higher in roots of seedlings treated with 10 µM CAN compared with the control, reaching 270 nmol g^−1^ FW. After application of 50 µM CAN for 24 h, the Put level in roots was about 150 nmol g^−1^ FW, which was similar to the control (Figure 5a).

After 72 h of the treatment, Put concentration in roots of control seedlings increased to approximately 250 nmol g^−1^ FW. In contrast, CAN supplementation led to a decrease in Put content. In seedlings treated with CAN, the Put concentration in roots was less than 50% of the control (Figure 5a).

The pattern of changes in Spd content in tomato roots exposed to CAN was similar to the pattern observed for Put (Figure 5a,b). After 24 h, Spd content was slightly higher in the roots of seedlings growing in 10 µM CAN compared with the control (Figure 5b). Exposure to 50 µM CAN did not alter Spd concentration, which was similar to that in the control, at the level of approximately 90 nmol g^−1^ FW.

After 72 h of culture, Spd concentration in control roots increased to 140 nmol g^−1^ FW. CAN resulted in a decrease in Spd content. In seedlings growing in 10 µM CAN, Spd concentration in roots decreased by 30% compared to the control, whereas exposure to CAN at a higher concentration resulted in a further decrease (Figure 5b).

Spm was the least abundant of the PAs in the tomato roots (Figure 5c). After 24 h of the treatment, Spm content in the control roots was low, reaching 6 nmol g^−1^ FW. CAN application resulted in a substantial increase in Spm concentration. In seedlings treated with 10 µM CAN, Spm content increased to 24 nmol g^−1^ FW, treatment with 50 µM CAN also led to elevated Spm levels, although the differences were not statistically significant. After 72 h, Spm concentration in the control roots remained at a level similar to that observed after 24 h. Prolonged CAN supplementation did not significantly alter Spm content in the roots of tomato seedlings (Figure 5c).

## 4. Discussion

In our previous studies, we have demonstrated that CAN inhibited Arg-dependent NO production and induced oxidative stress in tomato roots [23,26,27,28]. The present study shows that CAN strongly affects arginase activity, *ARG2* transcript levels, as well as disrupts PAs metabolism, suggesting that CAN induces a metabolic reprogramming of Arg utilisation. As Arg is a metabolite linking NO production, Orn formation, and PAs biosynthesis, CAN-induced interference with Arg metabolism may simultaneously affect these interconnected pathways. The changes in arginase activity observed in control seedlings are consistent with the developmental regulation of this enzyme. High arginase activity is typically associated with seed germination, when nitrogen reserves are intensively mobilised, whereas the transition to autotrophic growth is accompanied by a decline in enzyme activity as seed nitrogen reserves become depleted. A post-germinative decrease in arginase activity has previously been reported in Arabidopsis seedlings [51].

After 24 h of CAN exposure, arginase activity significantly decreased in tomato roots. CAN (at high, 5 mM concentration) itself could inhibit arginase activity as was demonstrated for arginase activity from murine and rat macrophages [52]. On the other hand, Herzfeld and Raper [53] showed that the kidney isoform of arginase could use CAN as a substrate. The plant arginase amino acid sequence is homologous to mammalian kidney-type arginases [54]. These observations were obtained in animal systems and at substantially higher CAN concentrations than those used in the present study.

The inhibition of arginase activity following short-term CAN treatment was accompanied by an increase in *ARG2* transcript level. The difference observed between *ARG2* transcript abundance and arginase activity may indicate the involvement of post-transcriptional regulatory mechanisms associated with CAN-induced nitro-oxidative imbalance. The low enzyme activity observed after 24 h of CAN treatment may result from post-translational modifications triggered by oxidative stress. Our previous studies demonstrated that short-term exposure to CAN increased the accumulation of carbonylated proteins in tomato roots [27]. Moreover, arginase activity depends on the integrity of its Mn^2+^-containing catalytic centre, which may be particularly susceptible to oxidative disturbances [55]. Oxidation of manganese from Mn^2+^ to Mn^4+^ has been shown to markedly reduce arginase activity [56]. In mammalian systems, arginase activity is regulated by oxidative and nitrosative signals through post-translational modifications, including *S*-nitrosation [57]. Although analogous mechanisms have not yet been demonstrated directly in plants, the high structural and catalytic conservation between plant and mammalian arginases supports the possibility that plant arginases may undergo similar regulatory modifications [22]. Moreover, these observations provide a possible mechanistic context (the high structural and catalytic conservation between plant and mammalian arginases supports the possibility that plant arginases may undergo similar regulatory modifications [22]). Neither the oxidation state of the Mn-containing catalytic centre nor *S*-nitrosation of arginase was analysed in the present study, and their involvement in the response of tomato roots to CAN remains hypothetical.

Another possible explanation for the decreased arginase activity after short-term CAN treatment may involve the formation of *N*^G^-hydroxy-L-canavanine. It has been proposed that oxidation of CAN by NOS yields *N*^G^-hydroxy-L-canavanine, which is subsequently decomposed to homoserine [58]. In mammals, the intermediate product of Arg oxidation by NOS, *N*^G^-hydroxy-L-arginine (NOHA), is a potent inhibitor of arginase activity [59]. Furthermore, NOHA has also been shown to inhibit arginase isolated from tomato leaves [25]. Therefore, *N*G-hydroxy-L-canavanine produced during CAN metabolism may hypothetically exert a similar inhibitory effect, contributing to the decreased arginase activity observed after 24 h of thetreatment. This proposed mechanism would provide an additional relationship between CAN interference with Arg-dependent NO metabolism and the regulation of arginase activity.

The high content of *ARG2* transcript levels observed just after 24 h of CAN treatment suggests that this isoform may participate in stress responses. Similar induction of arginase genes has been reported during wounding and jasmonate signalling, linking arginase activity to oxidative burst-associated defence responses [25]. Thus, elevated *ARG2* transcript level may represent a compensatory transcriptional response to maintain Arg catabolism and to restore Arg homeostasis despite reduced enzyme activity.

Arginase catalyses the hydrolysis of both Arg and CAN, yielding ornithine or canaline, respectively, together with urea, thereby contributing to nitrogen remobilisation and detoxification of excess CAN [1]. Although the enzyme displays a considerably higher affinity for Arg (Km 7–8 mM) than for CAN (Km 38 mM), as was demonstrated in CAN-producing legumes [60]. One possible, but untested, explanation is that increased arginase capacity could participate in CAN catabolism. Thus, the stimulation of arginase activity observed after prolonged exposure to 50 µM CAN may be considered as part of a detoxification response. Moreover, the relatively low Orn concentration after 72 h culture, despite elevated arginase activity, is consistent with the hypothesis that a proportion of enzyme activity is directed towards CAN hydrolysis rather than Arg catabolism.

Plants synthesise Orn from L-glutamate (Glu) or from Arg breakdown [61]. Glu can be synthesised via glutamine (Gln) synthetase (GS)/Glu synthase (also known as Gln-α-ketoglutarate aminotransferase, GOGAT) cycle in the chloroplasts of photosynthetic tissue or non-photosynthetic tissue plastids and Glu dehydrogenase (GDH) in the mitochondria or cytoplasm [62]. In our experiment, plants were grown in water, without an exogenous source of nitrogen. Thus, we suppose that the Orn synthesis after 72 h of seedling culture was rather based on Arg catabolism than derived from Glu. Moreover, Glu in germinating seeds and young developing seedlings originates mainly from degradation of stored proteins, so after prolonged experiment (72 h), we expected rather a deficiency of Glu and therefore its non-use as a substrate for Orn biosynthesis, which may explain the general decline in Orn concentration in both CAN-stressed and control roots.

CAN, a structural analogue of Arg, and canaline (a product of CAN hydrolysis by arginase), a structural analogue of Orn, inhibits Put biosynthesis because they are nonspecific inhibitors of ADC and ODC [63,64]. In our experiment, transcript levels of ODC were lowered (Figure S2), particularly by application of CAN at the higher dose just after 24 h, suggesting inhibition of Put synthesis. It corresponds well with the content of Put in tomato roots, which, after a transient increase in roots of seedlings exposed to 10 µM CAN for 24 h, declined about twice as CAN treatment was prolonged. Put was the most abundant among the tested PAs, significantly influencing the total content of these compounds in tomato roots. It is in agreement with the data obtained by Davis [65] on the hypocotyls of leafy spurge (*Euphorbia esula* L.), reporting the reduced content of free PAs in tissues exposed to CAN or canaline (at a concentration of 60 µM, similar to that used in our study). The comparable results were also shown by Palavan-Ünsal [66] on 5-day-old bean (*Phaseolus vulgaris* L.) roots grown in the presence of CAN. In tomato roots, as was described in segments of leafy spurge hypocotyls [65], 72 h long treatment with 50 µM CAN resulted in Put and Spd decline for about 50% as compared to the control. In contrast, Spm concentration in roots was elevated by short-term CAN stress, while its prolongation reduced Spm to the level of the control, non-stressed tissue. It is well known that Put content affects root growth and development [32,67]. Silencing of both *ADC1* and *ADC2* genes in Arabidopsis plants, in which Put biosynthesis depends only on ADC activity, showed a stunted growth of double mutants with a significant reduction in primary root length [68]. Prolonged (72 h) treatment with CAN of tomato seedlings resulted not only in lowering of Put and Spd levels but also in accumulation of H_2_O_2_ [27]. This is in agreement with the observation on Arabidopsis in which Put depletion led to the accumulation of H_2_O_2_ linked to PAO activity [68] rather than to stimulation of NADPH oxidase [27]. Also, in our material, both PAO activity and *PAO1* transcript levels were elevated, particularly after 24 h of CAN stress (Table S3, Figure S3). PAO activity and diamine oxidase activity were rapidly (within 1–3 h) stimulated in roots of cucumber (*Cucumis sativus* L.) treated with typical allelochemicals such as phenolic acids (ferulic, *p*-cumaric, *p*-hydroxybenzoic, and vanillic) at a concentration of 0.5 mM [69]. This was accompanied by the decreased Put and a drastic drop in Spd concentration. It is well established that PAs are involved in plant responses to various biotic and abiotic stresses and may contribute to the protection of plant cells against stress-induced injury. Moreover, changes in PAs biosynthesis and content differ depending on stress factors and tissue or developmental stage of the plant [70,71]. An application of PAs, which is expected to increase endogenous PAs, has been widely used in many experiments before or during stresses to mitigate their harmful effects, although these successful results might not reflect a physiological role of PAs in plant natural defence response [70,72]. Allelopathy is considered as biotic stress, but it differs in biochemical, transcriptomic, and metabolomic basis from typical biotic stresses (plant–pathogen or plant–herbivores interaction), for which PAs action is relatively well described and reviewed [72]. Studies concerning PAs and the action of allelopathic compounds in plants are very rare [43,73]. Our data indicate a decline in total PAs concentration after prolonged roots exposure to CAN, although transcript levels encoding enzymes of PAs biosynthesis (*SAMDC*, *SPDS*, *SPMS*) were not significantly modified. The exception is the only transient increase in *SPMS* transcript levels, corresponding to slightly higher Spm relative abundance among the total PAs pool. This observation represents the typical secondary mode of action of CAN as an allelochemical. A classical allelopathic compound such as artemisinin (a naturally occurring sesquiterpene lactone), at a concentration drastically inhibiting root growth of lettuce (*Lactuca sativa* L.), corn (*Zea mays* L.), or pea (*Pisum sativum* L.), only slightly reduced the content of Put and Spd, with almost no influence on Spm [74]. On the other hand, exogenous PAs were used to alleviate the inhibitory effect of some allelopathic compounds similarly as was described when PAs were applied in pre-treatment procedures to plants exposed to abiotic stressors [35,71]. A protective function of Spd against cinnamic acid, manifested by induction of cellular enzymatic antioxidants, was shown in pea seedlings [43]. Although there is no unique pattern of changes in PAs content and metabolism in plants exposed to allelochemicals, there is no doubt, that PAs, like other growth regulators or signalling compounds, take part in plants reaction to allelochemicals.

## 5. Conclusions

The primary mode of action of NPAA—CAN is directly related to its structural similarity to Arg and includes its incorporation into proteins, alteration of arginase activity, and suppression of Arg-dependent NO synthesis. Our results demonstrate that in tomato roots, CAN affects arginase transcript levels and enzymatic activity, with an initial inhibition of enzyme activity followed by its strong activation during prolonged exposure. This time-dependent response may reflect a transition from the primary inhibitory effect of CAN (or CAN-induced oxidative imbalance) to an adaptive response, potentially involving CAN detoxification through its conversion to canaline and urea by arginase. Short-term CAN exposure impacted the composition of the PA pool, whereas prolonged treatment led to a marked decrease in Put and Spd levels, accompanied by changes in *ODC* and *PAO1* transcript levels. Thus, disruption in PAs metabolism is an important secondary mode of CAN action, potentially contributing to altered redox homeostasis and to inhibition of root growth. Importantly, these responses should not be considered separately. Arg connects NO production, arginase activity, and PAs biosynthesis, while changes in PAs metabolism may additionally affect ROS generation. Demonstrated in this study, disturbances induced by CAN advance our understanding of alterations in PAs metabolism as an important component of the mode of action of allelochemicals.

## Figures and Tables

**Table 1:** Percentage contribution of each PA and total PAs (Put + Spd + Spm) concentration in root extracts of tomato seedlings grown in water (control) or 10 or 50 µM CAN after 24 and 72 h of the culture. Percentage contributions of individual PAs are presented as descriptive values. Total PAs concentration is presented as means ± SD, n = 4. Letters indicate homogeneous groups determined by two-way ANOVA followed by Tukey’s HSD test at *p* ≤ 0.05.

	Period of Treatment					
	24 h			72 h		
	Control	CAN 10 µM	CAN 50 µM	Control	CAN 10 µM	CAN 50 µM
Put (%)	61.2 ± 7.4	65 ± 17.8	59.2 ± 15.2	60.9 ± 8.4	61.2 ± 4.8	67.6 ± 6.4
Spd (%)	37.2 ± 0.9	29.4 ± 8.2	35.1 ± 4.2	37.8 ± 5.6	35.8 ± 8.5	29.4 ± 4.1
Spm (%)	1.9 ± 0.8	5.8 ± 0.7	5.8 ± 1.8	1.4 ± 0.3	3.3 ± 1.2	3.0 ± 0.1
Total PAs (nmol g^−1^ FW)	312.7 ± 43.9 ^ab^	418.9 ± 95.3 ^b^	227.4 ± 20.5 ^a^	416.5 ± 49.8 ^b^	233.9 ± 20.1 ^a^	173.0 ± 7.3 ^a^

## Data Availability

Upon request, the data will be provided by the corresponding author.

## Supplementary Materials

The following supporting information can be downloaded at: https://www.mdpi.com/article/10.3390/biology15171541/s1, Figure S1: Tomato seedlings grown for 24 or 72 h in water (control) or CAN solutions (10 or 50 µM). Images of representative seedlings are presented; Figure S2: Expression levels of ODC in roots of tomato plants grown in water (control) or treated with 10 or 50 µM CAN after 24 and 72 h of the culture. Transcript levels were normalised to EF1α and calculated using the 2^−ΔΔCt^ method, with the corresponding control set to 1. Values are means ± SD, n = 4. Letters indicate homogeneous groups determined by two-way ANOVA followed by Tukey’s HSD test at *p* ≤ 0.05 [75]; Figure S3: Expression levels of genes encoding polyamine oxidases (*PAO1* and *PAO2*) in roots of tomato plants grown in water (control) or treated with 10 or 50 µM CAN after 24 and 72 h of the culture. Values are means ± SD, n = 4. Letters indicate homogeneous groups determined by two-way ANOVA followed by Tukey’s HSD test at *p* ≤ 0.05; Table S1: Primer sequences, amplicon sizes, and PCR amplification efficiencies used for qRT-PCR analysis; Table S2: Shapiro–Wilk test for normality and Levene’s test for homogeneity of variance for the analysed parameters; Table S3: Activity of PAO in root extracts of tomato seedlings grown in water (control) or treated with 10 or 50 µM CAN after 24 and 72 h of the culture. Values are relative activity ± SD (according to Krasuska et al. [27]). Letters (a–d) indicate homogenous groups determined after ANOVA and Tukey HSD test at *p* ≤ 0.05. Control value is normalised to 1.

## Abbreviations

The following abbreviations are used in this manuscript:

Arg	Arginine
ADC	Arg decarboxylase
CAN	Canavanine
dcSAM	Decarboxylated *S*-adenosylmethionine
GDH	Glutamate dehydrogenase
Gln	Glutamine
Glu	Glutamate
GOGAT	Glutamate synthase
GS	Glutamine synthetase
NOHA	*N*^G^-hydroxy-L-arginine
NO	Nitric oxide
NOS	Nitric oxide synthase
NPAA	Non-proteinogenic amino acid
ODC	Ornithine decarboxylase
Orn	Ornithine
PAs	Polyamines
PAO	Polyamine oxidase
Put	Putrescine
RNS	Reactive nitrogen species
ROS	Reactive oxygen species
SAM	*S*-adenosylmethionine
SAMDC	*S*-adenosyl-L-methionine decarboxylase
Spd	Spermidine
SPDS	Spermine synthase
Spm	Spermidine
SPMS	Spermine synthase

## References

[1] Rosenthal G.A. (1977). Nitrogen Allocation for L-Canavanine Synthesis and Its Relationship to Chemical Defense of the Seed. Biochem. Syst. Ecol..

[2] Schlüter M., Bordas E. (1972). Canavanine in *Canavalia paraguayensis*, *C. gladiata* and *Dioclea paraguayensis*. Phytochemistry.

[3] Staszek P., Weston L.A., Ciacka K., Krasuska U., Gniazdowska A. (2017). L-Canavanine: How Does a Simple Non-Protein Amino Acid Inhibit Cellular Function in a Diverse Living System?. Phytochem. Rev..

[4] Rosenthal G.A., Nkomo P. (2000). The Natural Abundance Of L-Canavanine, An Active Anticancer Agent, in Alfalfa, *Medicago sativa* (L.). Pharm. Biol..

[5] Miersch J., Jühlke C., Sternkopf G., Krauss G.J. (1992). Metabolism and Exudation of Canavanine during Development of Alfalfa (*Medicago sativa* L. Cv. Verko). J. Chem. Ecol..

[6] Soltys D., Gniazdowska A., Bogatek R. (2013). Inhibition of Tomato (*Solanum lycopersicum* L.) Root Growth by Cyanamide Is Not Always Accompanied with Enhancement of ROS Production. Plant Signal. Behav..

[7] Soltys D., Rudzińska-Langwald A., Kurek W., Gniazdowska A., Sliwinska E., Bogatek R. (2011). Cyanamide Mode of Action during Inhibition of Onion (*Allium cepa* L.) Root Growth Involves Disturbances in Cell Division and Cytoskeleton Formation. Planta.

[8] Soltys D., Rudzińska-Langwald A., Kurek W., Szajko K., Sliwinska E., Bogatek R., Gniazdowska A. (2014). Phytotoxic Cyanamide Affects Maize (*Zea mays*) Root Growth and Root Tip Function: From Structure to Gene Expression. J. Plant Physiol..

[9] Kamo T., Sakurai S., Yamanashi T., Todoroki Y. (2015). Cyanamide Is Biosynthesized from L-Canavanine in Plants. Sci. Rep..

[10] Sasamoto H., Mardani H., Sasamoto Y., Wasano N., Murashige-Baba T., Sato T., Hasegawa A., Fujii Y. (2019). Evaluation of Canavanine as an Allelochemical in Etiolated Seedlings of *Vicia villosa* Roth: Protoplast Co-Culture Method with Digital Image Analysis. In Vitro Cell. Dev. Biol.—Plant.

[11] Mardani-Korrani H., Nakayasu M., Yamazaki S., Aoki Y., Kaida R., Motobayashi T., Kobayashi M., Ohkama-Ohtsu N., Oikawa Y., Sugiyama A. (2021). L-Canavanine, a Root Exudate From Hairy Vetch (*Vicia villosa*) Drastically Affecting the Soil Microbial Community and Metabolite Pathways. Front. Microbiol..

[12] Ignacio M., Rodríguez-Robles D., Zambelli A., Nocito F.F., Araniti F. (2025). Unveiling the Multifaceted Roles of Root Exudates: Chemical Interactions, Allelopathy, and Agricultural Applications. Agronomy.

[13] Igloi G.L., Schiefermayr E. (2009). Amino Acid Discrimination by Arginyl-TRNA Synthetases as Revealed by an Examination of Natural Specificity Variants. FEBS J..

[14] Rosenthal G.A. (2001). L-Canavanine: A Higher Plant Insecticidal Allelochemical. Amino Acids.

[15] Dahlman D.L., Rosenthal G.A. (1975). Non-Protein Amino Acid-Insect Interactions—I. Growth Effects and Symptomology of l-Canavanine Consumption by Tobacco Hornworm, *Manduca sexta* (L.). Comp. Biochem. Physiol. A Physiol..

[16] Dahlman D.L., Rosenthal G.A. (1976). Further Studies of the Effect of L-Canavanine on the Tobacco Hornworm, *Manduca sexta*. J. Insect Physiol..

[17] Devambez I., Ali Agha M., Mitri C., Bockaert J., Parmentier M.-L., Marion-Poll F., Grau Y., Soustelle L. (2013). Gαo Is Required for L-Canavanine Detection in *Drosophila*. PLoS ONE.

[18] Vynnytska B.O., Mayevska O.M., Kurlishchuk Y.V., Bobak Y.P., Stasyk O. (2011). V Canavanine Augments Proapoptotic Effects of Arginine Deprivation in Cultured Human Cancer Cells. Anticancer Drugs.

[19] Bence A.K., Adams V.R., Crooks P.A. (2003). L-Canavanine as a Radiosensitization Agent for Human Pancreatic Cancer Cells. Mol. Cell. Biochem..

[20] Nurcahyanti A.D., Wink M. (2016). L-Canavanine Potentiates the Cytotoxicity of Doxorubicin and Cisplatin in Arginine Deprived Human Cancer Cells. PeerJ.

[21] Jubault M., Hamon C., Gravot A., Lariagon C., Delourme R., Bouchereau A., Manzanares-Dauleux M.J. (2008). Differential Regulation of Root Arginine Catabolism and Polyamine Metabolism in Clubroot-Susceptible and Partially Resistant Arabidopsis Genotypes. Plant Physiol..

[22] Winter G., Todd C.D., Trovato M., Forlani G., Funck D. (2015). Physiological Implications of Arginine Metabolism in Plants. Front. Plant Sci..

[23] Staszek P., Gniazdowska A. (2020). Peroxynitrite Induced Signaling Pathways in Plant Response to Non-Proteinogenic Amino Acids. Planta.

[24] Flores T., Todd C.D., Tovar-Mendez A., Dhanoa P.K., Correa-Aragunde N., Hoyos M.E., Brownfield D.M., Mullen R.T., Lamattina L., Polacco J.C. (2008). Arginase-Negative Mutants of Arabidopsis Exhibit Increased Nitric Oxide Signaling in Root Development. Plant Physiol..

[25] Chen H., McCaig B.C., Melotto M., He S.Y., Howe G.A. (2004). Regulation of Plant Arginase by Wounding, Jasmonate, and the Phytotoxin Coronatine. J. Biol. Chem..

[26] Krasuska U., Andrzejczak O., Staszek P., Borucki W., Gniazdowska A. (2016). Toxicity of Canavanine in Tomato (*Solanum lycopersicum* L.) Roots Is Due to Alterations in RNS, ROS and Auxin Levels. Plant Physiol. Biochem..

[27] Krasuska U., Andrzejczak O., Staszek P., Bogatek R., Gniazdowska A. (2016). Canavanine Alters ROS/RNS Level and Leads to Post-Translational Modification of Proteins in Roots of Tomato Seedlings. Front. Plant Sci..

[28] Staszek P., Krasuska U., Otulak-Kozieł K., Fettke J., Gniazdowska A. (2019). Canavanine-Induced Decrease in Nitric Oxide Synthesis Alters Activity of Antioxidant System but Does Not Impact S-Nitrosoglutathione Catabolism in Tomato Roots. Front. Plant Sci..

[29] Kolbert Z., Wendehenne D., Boscari A. (2026). Current Directions in Plant Nitric Oxide Research. Plant Sci..

[30] Krasuska U., Ciacka K., Orzechowski S., Fettke J., Bogatek R., Gniazdowska A. (2016). Modification of the Endogenous NO Level Influences Apple Embryos Dormancy by Alterations of Nitrated and Biotinylated Protein Patterns. Planta.

[31] Wang W., Paschalidis K., Feng J.C., Song J., Liu J.H. (2019). Polyamine Catabolism in Plants: A Universal Process with Diverse Functions. Front. Plant Sci..

[32] González-Hernández A.I., Scalschi L., Vicedo B., Marcos-Barbero E.L., Morcuende R., Camañes G. (2022). Putrescine: A Key Metabolite Involved in Plant Development, Tolerance and Resistance Responses to Stress. Int. J. Mol. Sci..

[33] Alabadí D., Carbonell J. (1998). Expression of Ornithine Decarboxylase Is Transiently Increased by Pollination, 2,4-Dichlorophenoxyacetic Acid, and Gibberellic Acid in Tomato Ovaries. Plant Physiol..

[34] Hanfrey C., Sommer S., Mayer M.J., Burtin D., Michael A.J. (2001). Arabidopsis Polyamine Biosynthesis: Absence of Ornithine Decarboxylase and the Mechanism of Arginine Decarboxylase Activity. Plant J..

[35] Chen D., Shao Q., Yin L., Younis A., Zheng B. (2019). Polyamine Function in Plants: Metabolism, Regulation on Development, and Roles in Abiotic Stress Responses. Front. Plant Sci..

[36] Zhu M., Chen G., Wu J., Wang J., Wang Y., Guo S., Shu S. (2023). Identification of Cucumber S-Adenosylmethionine Decarboxylase Genes and Functional Analysis of CsSAMDC3 in Salt Tolerance. Front. Plant Sci..

[37] Jia T., Hou J., Iqbal M.Z., Zhang Y., Cheng B., Feng H., Li Z., Liu L., Zhou J., Feng G. (2021). Overexpression of the White Clover TrSAMDC1 Gene Enhanced Salt and Drought Resistance in *Arabidopsis thaliana*. Plant Physiol. Biochem..

[38] Kumar A., Taylor M.A., Mad Arif S.A., Davies H.V. (1996). Potato Plants Expressing Antisense and Sense S-Adenosylmethionine Decarboxylase (SAMDC) Transgenes Show Altered Levels of Polyamines and Ethylene: Antisense Plants Display Abnormal Phenotypes. Plant J..

[39] Bogatek R., Gniazdowska A. (2007). ROS and Phytohormones in Plant-Plant Allelopathic Interaction. Plant Signal. Behav..

[40] Gniazdowska A., Krasuska U., Andrzejczak O., Soltys D., Gupta K.J., Igamberdiev A.U. (2015). Allelopathic Compounds as Oxidative Stress Agents: Yes or NO. Reactive Oxygen and Nitrogen Species Signaling and Communication in Plants, Signaling and Communication in Plants.

[41] Šoln K., Klemenčič M., Koce J.D. (2022). Plant Cell Responses to Allelopathy: From Oxidative Stress to Programmed Cell Death. Protoplasma.

[42] Staszek P., Krasuska U., Wal A., Zak J., Gniazdowska A. (2022). NO and Metabolic Reprogramming under Phytotoxicity Stress. Nitric Oxide in Plant Biology: An Ancient Molecule with Emerging Roles.

[43] Kapoor R.T., Alyemeni M.N., Ahmad P. (2021). Exogenously Applied Spermidine Confers Protection against Cinnamic Acid-Mediated Oxidative Stress in *Pisum sativum*. Saudi J. Biol. Sci..

[44] Shao J., Huang K., Batool M., Idrees F., Afzal R., Haroon M., Noushahi H.A., Wu W., Hu Q., Lu X. (2022). Versatile Roles of Polyamines in Improving Abiotic Stress Tolerance of Plants. Front. Plant Sci..

[45] Liu J.H., Wang W., Wu H., Gong X., Moriguchi T. (2015). Polyamines Function in Stress Tolerance: From Synthesis to Regulation. Front. Plant Sci..

[46] Labudda M., Różańska E., Cieśla J., Sobczak M., Dzik J.M. (2016). Arginase Activity in *Arabidopsis thaliana* Infected with *Heterodera schachtii*. Plant Pathol..

[47] Olson B.J.S.C., Markwell J. (2007). Assays for Determination of Protein Concentration. Curr. Protoc. Pharmacol..

[48] Krasuska U., Ciacka K., Gniazdowska A. (2017). Nitric Oxide-Polyamines Cross-Talk during Dormancy Release and Germination of Apple Embryos. Nitric Oxide.

[49] Ruijter J.M., Ramakers C., Hoogaars W.M.H., Karlen Y., Bakker O., Van den Hoff M.J.B., Moorman A.F.M. (2009). Amplification Efficiency: Linking Baseline and Bias in the Analysis of Quantitative PCR Data. Nucleic Acids Res..

[50] Hellemans J., Mortier G., De Paepe A., Speleman F., Vandesompele J. (2007). QBase Relative Quantification Framework and Software for Management and Automated Analysis of Real-Time Quantitative PCR Data. Genome Biol..

[51] Zonia L.E., Stebbins N.E., Polacco J.C. (1995). Essential Role of Urease in Germination of Nitrogen-Limited *Arabidopsis thaliana* Seeds. Plant Physiol..

[52] Hrabåk A., Bajor T., Temesi Å. (1994). Comparison of Substrate and Inhibitor Specificity of Arginase and Nitricm Oxide (NO) Synthase for Arginine Analogs and Related Compounds in Murine and Rat Macrophages. Biochem. Biophys. Res. Commun..

[53] Herzfeld A., Raper S.M. (1976). The Heterogeneity of Arginases in Rat Tissues. Biochem. J..

[54] Siddappa S., Marathe G.K. (2020). What We Know about Plant Arginases?. Plant Physiol. Biochem..

[55] Cox J.D., Cama E., Colleluori D.M., Pethe S., Boucher J.-L., Mansuy D., Ash D.E., Christianson D.W. (2001). Mechanistic and Metabolic Inferences from the Binding of Substrate Analogues and Products to Arginase. Biochemistry.

[56] Berüter J., Colombo J.P., Bachmann C. (1978). Purification and Properties of Arginase from Human Liver and Erythrocytes. Biochem. J..

[57] Santhanam L., Lim H.K., Lim H.K., Miriel V., Brown T., Patel M., Balanson S., Ryoo S., Anderson M., Irani K. (2007). Inducible NO Synthase-Dependent S-Nitrosylation and Activation of Arginase1 Contribute to Age-Related Endothelial Dysfunction. Circ. Res..

[58] Luzzi S.D., Marletta M.A. (2005). L-Arginine Analogs as Alternate Substrates for Nitric Oxide Synthase. Bioorg. Med. Chem. Lett..

[59] Daghigh F., Fukuto J.M., Ash D.E. (1994). Inhibition of Rat Liver Arginase by an Intermediate in NO Biosynthesis, NG-Hydroxy-L-Arginine: Implications for the Regulation of Nitric Oxide Biosynthesis by Arginase. Biochem. Biophys. Res. Commun..

[60] Downum K.R., Rosenthal G.A., Cohen W.S. (1983). L-Arginine and L-Canavanine Metabolism in Jack Bean, *Canavalia Ensiformis* (L.) DC. and Soybean, *Glycine Max* (L.) Merr. Plant Physiol..

[61] Majumdar R., Barchi B., Turlapati S.A., Gagne M., Minocha R., Long S., Minocha S.C. (2016). Glutamate, Ornithine, Arginine, Proline, and Polyamine Metabolic Interactions: The Pathway Is Regulated at the Post-Transcriptional Level. Front. Plant Sci..

[62] Bernard S.M., Habash D.Z. (2009). The Importance of Cytosolic Glutamine Synthetase in Nitrogen Assimilation and Recycling. New Phytol..

[63] Altman A., Bachrach U. (1981). Involvement of Polyamines in Plant Growth and Senescence. Adv. Polyam. Res..

[64] Mengoli M., Bagni N., Luccarini G., Ronchi V.N., Serafini-Fracassini D. (1989). *Daucus carota* Cell Cultures: Polyamines and Effect of Polyamine Biosynthesis Inhibitors in the Preembryogenic Phase and Different Embryo Stages. J. Plant Physiol..

[65] Davis D.G. (1997). Polyamines, Auxins and Organogenesis in Leafy Spurge (*Euphorbia esula* L.). J. Plant Physiol..

[66] Palavan-ünsal N. (1987). Polyamine Metabolism in the Roots of *Phaseolus vulgaris*. Interaction of the Inhibitors of Polyamine Biosynthesis with Putrescine in Growth and Polyamine Biosynthesis. Plant Cell Physiol..

[67] Hashem A.M., Moore S., Chen S., Hu C., Zhao Q., Elesawi I.E., Feng Y., Topping J.F., Liu J., Lindsey K. (2021). Putrescine Depletion Affects Arabidopsis Root Meristem Size by Modulating Auxin and Cytokinin Signaling and ROS Accumulation. Int. J. Mol. Sci..

[68] Sánchez-Rangel D., Chávez-Martínez A.I., Rodríguez-Hernández A.A., Maruri-López I., Urano K., Shinozaki K., Jiménez-Bremont J.F. (2016). Simultaneous Silencing of Two Arginine Decarboxylase Genes Alters Development in Arabidopsis. Front. Plant Sci..

[69] Politycka B., Kubis J. (2000). Changes in Free Polyamine Level and Di- and Polyamine Oxidase Activity in Cucumber Roots under Allelochemical Stress Conditions. Acta Physiol. Plant..

[70] Blázquez M.A. (2024). Polyamines: Their Role in Plant Development and Stress. Annu. Rev. Plant Biol..

[71] Minocha R., Majumdar R., Minocha S.C. (2014). Polyamines and Abiotic Stress in Plants: A Complex Relationship. Front. Plant Sci..

[72] Nawaz M., Shabbir S., Sonne C., Xiaobo L. (2026). Polyamine Defense Shield of Plants against Biotic and Abiotic Stress. Plant Stress.

[73] Huang X., Bie Z. (2010). Cinnamic Acid-Inhibited Ribulose-1,5-Bisphosphate Carboxylase Activity Is Mediated through Decreased Spermine and Changes in the Ratio of Polyamines in Cowpea. J. Plant Physiol..

[74] DiTomaso J.M., Duke S.O. (1991). Is Polyamine Biosynthesis a Possible Site of Action of Cinmethylin and Artemisinin?. Pestic. Biochem. Physiol..

[75] Krasuska U., Andrzejczak O., Staszek P., Borucki W., Gniazdowska A. (2017). Meta-Tyrosine Induces Modification of Reactive Nitrogen Species Level, Protein Nitration and Nitrosoglutathione Reductase in Tomato Roots. Nitric Oxide.

