# Supplementary material for: Alterations in Arginase Activity and Polyamine Metabolism Contribute to the Mode of Action of Canavanine in Tomato Roots
Source: Biology (Basel). 2026 Sep 4;15(17):1541. doi: 10.3390/biology15171541 (PMC13564764; doi:10.3390/biology15171541)
Supplement: Supplementary file 1 [file biology-15-01541-s001.zip › biology-4487476-supplementary.pdf]

Figure S1. Tomato seedlings grown for 24 or 72 h in water (control) or CAN solutions (10 or 50 μM). Images of representative seedlings are presented. The white balance was adjusted using GenAI.

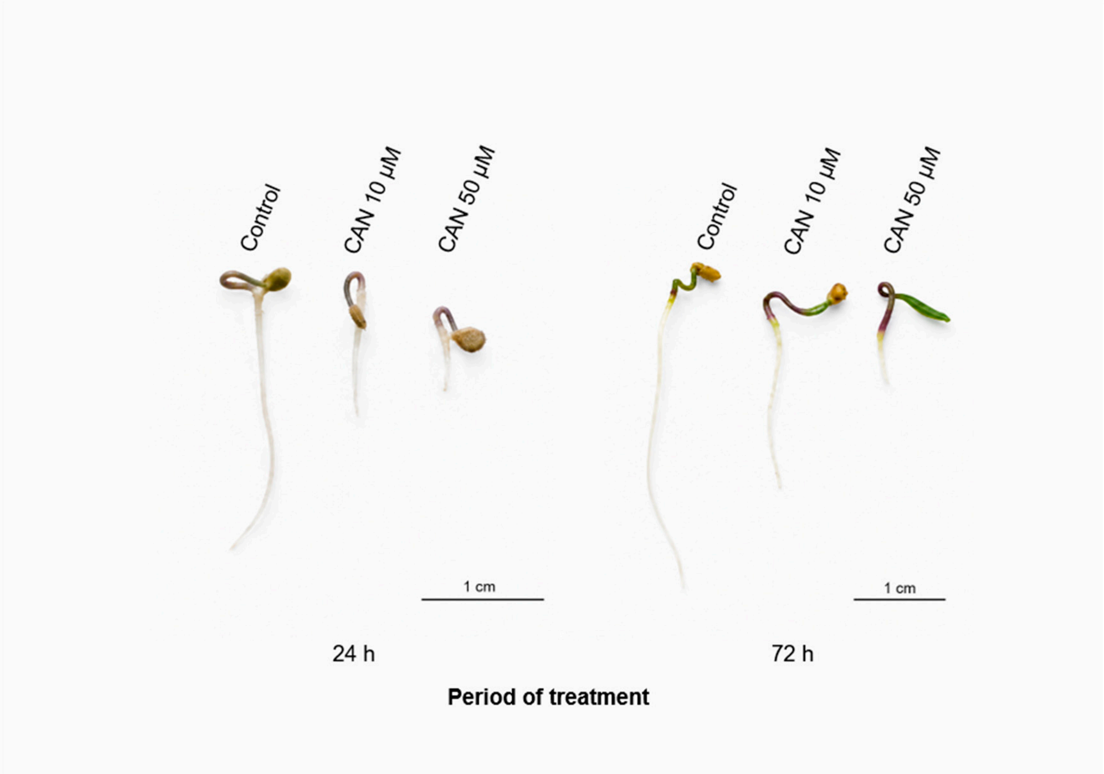

Table S1. Primer sequences, amplicon sizes, and PCR amplification efficiencies used for qRT-PCR analysis

Gene	NCBI accession number	Forward primer	Reverse primer	Amplicon size (bp)	PCR efficiency (%)
ARG1	NM_001247649.2	GCTTTGCACGGATAATGGAG	ATATTGCTCCACACCGAACC	113	100.8
ARG2	NM_001246922.2	AGCGTCAACGTCACTTCTTG	AAGAGGAGGAGCAAATGCAG	79	101.6
SAMDC	NM_001247770.3	GCAGAAACTGGCAAGTCCAT	CACAAAGGCACAGTTGGATG	133	108.0
SPDS	NM_001247564.4	GAATCTCCACATTGGTGACG	AAACAACTCTTGCGCTGGAC	112	101.0
SPMS	NM_001247478.1	AACATGGCAGAGAGCATGTG	AACCCAATAACGCCACTAGG	131	105.0
ODC	NM_001247687.1	AAACCCACTTCCACGACTTC	GACTCTTTTGCCGATGATGG	107	107.0
PAO1	XM_004229603.5	CCGGAATCTCAGGATTAACG	TATCAGCCGCCTCCAATATC	81	104.0
PAO2	XM_004243582.5	GGCCCATGACATTGAGAAAC	GCATTGGGAAGAATCCTCTG	81	106.0
EF1α	XM_004240531.4	GGTCATCATCATGAACCATCC	CATACCAGCATCACCGTTCTT	175	100.8
PP2Acs	NM_001247587.2	CGATGTGTGATCTCCTATGGTC	AAGCTGATGGGCTCTAGAAATC	149	91.6

Table S2. Shapiro–Wilk test for normality and Levene's test for homogeneity of variance for the analysed parameters

Parameter	Shapiro–Wilk *p*-value	Levene *p*-value
Arginase activity	0.963	0.514
ARG1	0.408	0.305
ARG2	0.999	0.726
Orn	0.563	0.334
SAMDC	0.251	0.421
SPDS	0.373	0.720
SPMS	0.581	0.909
Put	0.661	0.482
Spd	0.253	0.604
Spm	0.834	0.704
ODC	0.433	0.082
PAO1	0.921	0.160
PAO2	0.175	0.830

Analysis of transcript levels of gene encoding ODC

cDNA synthesis and qRT-PCR were performed as described in Section 2.4. of the Materials and Methods in the main manuscript, except that EF1α was used as the reference gene [75]. Relative *ODC* transcript levels were calculated using the $2^{-\Delta\Delta Ct}$ method, normalised to *EF1*α, and calibrated to the corresponding control at each treatment duration (control = 1). For statistical analysis, ΔCt values of individual biological replicates were analysed by two-way ANOVA, followed by Tukey's HSD test at $p \leq 0.05$.

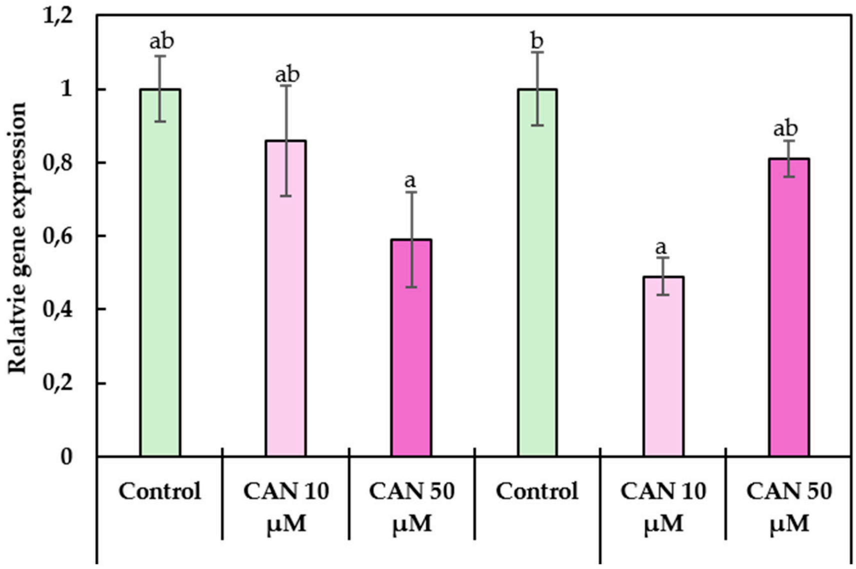

Figure S2. Expression levels of *ODC* in roots of tomato plants grown in water (control) or treated with 10 or 50 µM CAN after 24 and 72 h of the culture. Transcript levels were normalised to EF1α and calculated using the $2^{-\Delta\Delta Ct}$ method, with the corresponding control set to 1. Values are means ± SD, n=4. Letters indicate homogeneous groups determined by two-way ANOVA followed by Tukey's HSD test at $p \leq 0.05$.After 24 h, expression of *ODC* in roots of plants treated with 10 µM CAN was slightly lower than in the control, whereas supplementation with 50 µM CAN resulted in a stronger downregulation of *ODC* (Fig.S2). After 72 h of the culture, *ODC* transcript levels were decreased in all treated plants. The significant inhibition was observed after exposure to 10 µM CAN. CAN at higher dose significantly reduced *ODC* transcript levels, although expression remained higher than that recorded in roots of plants treated with 10 µM CAN

Analysis of transcript levels of genes encoding PAO

cDNA synthesis and qRT-PCR and calculations were performed as described in Section 2.4. of the Materials and Methods in the main manuscript.

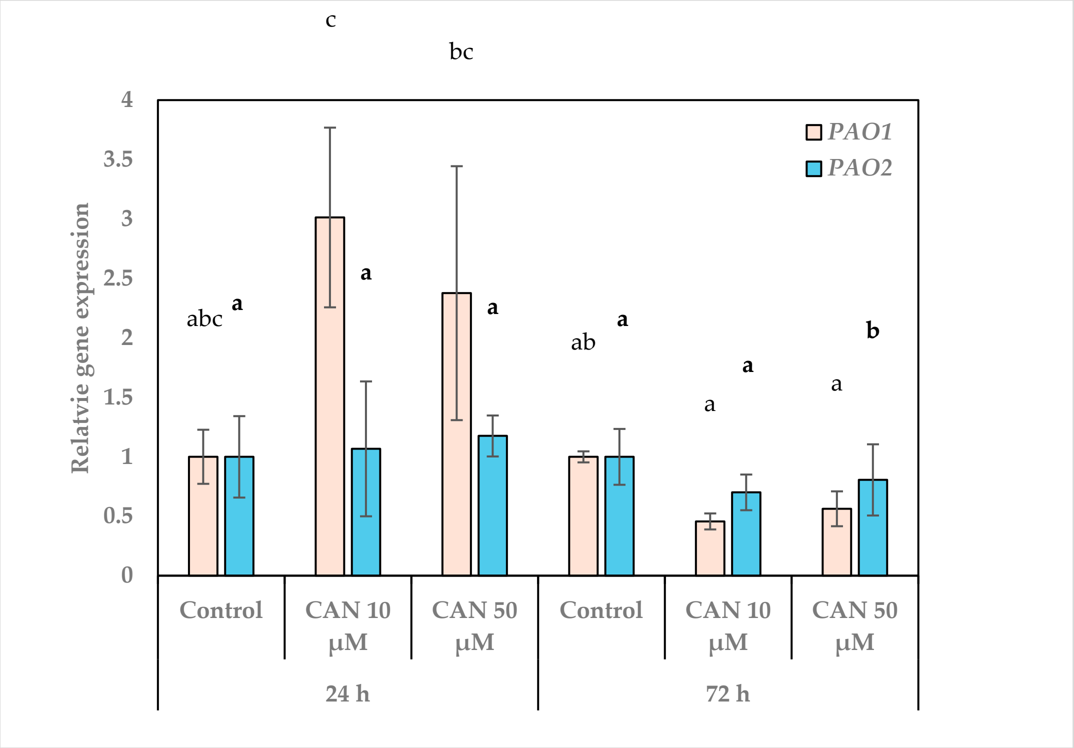

Figure S3. Expression levels of genes encoding polyamine oxidases (PAO1 and PAO2) in roots of tomato plants grown in water (control) or treated with 10 or 50 µM CAN after 24 and 72 h of the culture. Values are means ± SD, n = 4. Letters indicate homogeneous groups determined by two-way ANOVA followed by Tukey's HSD test at $p \leq 0.05$.

Transcript levels of genes encoding proteins related to PAs catabolism, including (*PAO1*, *PAO2*), were analysed in roots of tomato seedlings after 24 and 72 h of CAN treatment (Fig. S3). After 24 h of the culture, *PAO1* transcript levels were elevated in roots treated with both CAN concentrations. In contrast, after 72 h *PAO1* transcript levels were lower in roots of seedlings treated with 10 µM or 50 µM CAN. Transcript levels of *PAO2* were higher in roots of tomato seedlings treated with 50 µM CAN, whereas treatment with 10 µM CAN did not significantly affect *PAO2* transcript abundance.

PAO activity

The data of PAO activity obtained under the same experimental conditions were previously published by Krasuska et al. [27]. Therefore, the relative PAO activity is shown in Table S1, with the control value normalised to 1.

Table S3. Activity of PAO in root extracts of tomato seedlings grown in water (control) or treated with 10 or 50 μM CAN after 24 and 72 h of the culture. Values are relative activity ± SD (according to Krasuska et al. [27]). Letters (a-d) indicate homogenous groups determined after ANOVA and Tukey HSD test at $p \leq 0.05$. Control value is normalised to 1

	Activity of PAO (relative units)	
	24 h	72 h
Control	1 ± 0.09^{a}	1 ± 0.09^{a}
CAN 10 μM	1.55 ± 0.26^{b}	1.88 ± 0.38^{c}
CAN 50 μM	1.60 ± 0.9^{b}	2.94 ± 0.44^{d}

After 24 h of treatment, CAN increased PAO activity approximately 1.6-fold at both concentrations compared with the control. After 72 h, the stimulatory effect was more pronounced, reaching approximately 1.9- and 2.9-fold of the control in roots of plants treated with 10 and 50 μM CAN, respectively.
